# Application of triple evaluation method in predicting the efficacy of neoadjuvant therapy for breast cancer

**DOI:** 10.1186/s12957-023-02998-8

**Published:** 2023-03-29

**Authors:** Xu Han, Hui Li, Sha-Sha Dong, Shui-Ying Zhou, Cai-Hong Wang, Lin Guo, Jie Yang, Gang-Ling Zhang

**Affiliations:** 1 Department of Breast Surgery, Baotou Cancer Hospital, No.18 Tuanjie Street, Qingshan District, Baotou, 014030 Inner Mongolia China; 2 Department of Operating Room, Baotou Cancer Hospital, Baotou, 014030 Inner Mongolia China

**Keywords:** Breast cancer, Neoadjuvant therapy, Triple evaluation method, Physical examination, Color Doppler ultrasound, Magnetic resonance imaging

## Abstract

**Objective:**

To analyze the factors related to the efficacy of neoadjuvant therapy for breast cancer and find appropriate evaluation methods for evaluating the efficacy of neoadjuvant therapy

**Methods:**

A total of 143 patients with breast cancer treated by neoadjuvant chemotherapy at Baotou Cancer Hospital were retrospectively analyzed. The chemotherapy regimen was mainly paclitaxel combined with carboplatin for 1 week, docetaxel combined with carboplatin for 3 weeks, and was replaced with epirubicin combined with cyclophosphamide after evaluation of disease progression. All HER2-positive patients were treated with simultaneous targeted therapy, including trastuzumab single-target therapy and trastuzumab combined with pertuzumab double-target therapy. Combined with physical examination, color Doppler ultrasound, and magnetic resonance imaging (MRI), a systematic evaluation system was initially established—the “triple evaluation method.” A baseline evaluation was conducted before treatment. The efficacy was evaluated by physical examination and color Doppler every cycle, and the efficacy was evaluated by physical examination, color Doppler, and MRI every two cycles.

**Results:**

The increase in ultrasonic blood flow after treatment could affect the efficacy of monitoring. The presence of two preoperative time–signal intensity curves is a therapeutically effective protective factor for inflow. The triple evaluation determined by physical examination, color Doppler ultrasound, and MRI in determining clinical efficacy is consistent with the effectiveness of the pathological gold standard.

**Conclusion:**

The therapeutic effect of neoadjuvant therapy can be better evaluated by combining clinical physical examination, color ultrasound, and nuclear magnetic resonance evaluation. The three methods complement each other to avoid the insufficient evaluation of a single method, which is convenient for most prefecty-level hospitals. Additionally, this method is simple, feasible, and suitable for promotion.

## Introduction

Neoadjuvant therapy has become an important method in the treatment of breast cancer. Patricia et al. confirmed that after neoadjuvant therapy, the patient can achieve better event-free survival (EFS) and overall survival (OS) in the pathological complete response (pCR), especially in triple-negative breast cancer (EFS: HR0.24; OS: 0.16) and HR-negative HER2-positive breast cancer (EFS: 0.15; OS: 0.08) [1]. The Expert Consensus on Neoadjuvant Therapy for Breast Cancer in China (2022 edition) highlights that understanding tumor reactivity to the corresponding treatment using neoadjuvant therapy and formulating the follow-up adjuvant therapy strategy based on whether the pCR is achieved after the whole course of neoadjuvant therapy is vital for patients who are to undergo neoadjuvant therapy [2]. All guidelines recommend that to ensure the efficacy of neoadjuvant therapy, patients should undergo weekly color Doppler ultrasound examination and MRI every two cycles [3, 4]. However, there is no uniform standard for the selection of specific parameters, and only the response evaluation criteria in solid tumors (RECIST), that is, the maximum diameter changes of target lesions before and after measurement, is used to evaluate the efficacy [3–7]. The American Society of Radiology recommends breast MRI as the highest grade for baseline examination, mid-treatment, and post-treatment evaluation of breast cancer and breast ultrasound as the highest grade for pre- and post-treatment axillary lymph node evaluation [8]. Although ultrasound alone is not more beneficial compared with MRI, existing research also indicates that there is no statistical difference between ultrasound and MRI in aspects such as sensitivity, negative predictive value, and accuracy [9]. Based on the meta-analysis conducted by Samiei et al., most commonly used non-invasive clinical examination methods can only be used to measure the size of axillary lymph node positive lesions and lack sensitivity to evaluate the pCR status of lymph node metastases post-treatment [10, 11]. As the relationship between axillary status and efficacy of breast lesions is still unclear, and further exploration is needed, we only explored the evaluation method of refining target breast lesions. At present, studies on the predictors of the efficacy of neoadjuvant chemotherapy (NAC) for breast cancer include body mass index, tumor size, molecular subtype, peripheral blood neutrophil-lymphocyte ratio, and tumor-infiltrating lymphocytes [12, 13]. Long-chain non-coding RNA, tumor circulating DNA, and multiple gene classification stages have also become hot spots in prediction research in recent years [14, 15].

At present, it is found in clinical work that there are certain limitations in evaluating the therapeutic response by NMR, color ultrasound, or physical examination, and the research on the combination of the three methods is extremely limited.

Therefore, in this study, combined with physical examination, color Doppler ultrasound, and magnetic resonance imaging (MRI), there was a preliminary formation of breast cancer new adjuvant therapy system evaluation system, namely the triple evaluation method. The purpose of this study was to analyze the value of triple evaluation in predicting response to neoadjuvant treatment.

## Data and methods

### Study participants

This study was approved by the Ethics Committee of Baotou Cancer Hospital. This study was conducted in accordance with the Declaration of Helsinki. Written informed consent was obtained from all the participants.

A total of 143 patients with breast cancer treated at the Department of Breast Cancer, Baotou Cancer Hospital from January 2018 to December 2021 were analyzed. All patients underwent mass puncture procedure and their immunohistochemical results were obtained. The clinical stages of the patients ranged from stage II to III. All patients were confirmed by puncture biopsy, and immunochemotherapeutic tests were performed (meaning that patients had a clear diagnosis and immunohistochemical type prior to treatment). All enrolled patients with breast cancer underwent neoadjuvant chemotherapy and completed six to eight cycles of therapy. The physical examination, color Doppler ultrasound, and MRI data were recorded at baseline prior to commencing treatment; the physical examination and color Doppler ultrasound data were recorded every cycle; and the physical examination, color Doppler ultrasound, and MRI data were recorded every two cycles to evaluate the therapeutic effect. Finally, the surgical procedures were performed in the hospital and postoperative pathological results were obtained.

The chemotherapy regimen was mainly paclitaxel combined with carboplatin for 1 week and docetaxel combined with carboplatin for 3 weeks and was replaced with epirubicin combined with cyclophosphamide after evaluation of disease progression. All HER2-positive patients were treated with simultaneous targeted therapy, including trastuzumab single-target and trastuzumab combined with pertuzumab double-target therapy.

### Imaging examinations and methods

Color Doppler ultrasound (US) examination method: Resona R9 and Philips IU22 color Doppler ultrasound instruments were used, with a probe frequency of 5–10 MHz. The patients were placed in a supine position, to fully expose the chest and armpits, and both hands were placed on top of the head. The position, shape, size, edge, internal echo, calcification, calcification type, and boundary with the surrounding tissues were observed using conventional color Doppler ultrasound, and the volume of the tumor (length×width×thickness) was measured. Color Doppler was used to display the blood flow signal distribution and shape in the mass, and the blood flow resistance index and peak blood flow rate were measured. The angle was corrected during measurement; the corrected angle was less than 60° and the angle was 0° for punctate blood flow. The elasticity of the tumor was graded using shear wave elastography. Simultaneously, the morphology and structure of the bilateral axillary lymph nodes were examined to determine whether enlarged or plump lymph nodes with thickened cortices were present.

The color Doppler flow signal was obtained by referring to the Adler flow classification, which is divided into four grades based on the blood flow morphology and distribution inside the tumor: Grade 0, no flow signal is found in the mass; Grade I, a small amount of blood flow and one to two punctiform or thin rod-shaped tumor vessels are visible, with rod-shaped blood flow not exceeding 1/2 of the diameter of the lesion; Grade II, medium (accessible) blood flow, three to four punctiform blood vessels or a long vessel penetrating the lesion are visible, the length of which could be close to or exceeding the radius of the mass; and Grade III, massive (rich) blood flow, with ≥ 5 punctiform vessels or 2 longer vessels visible. The flow enrichment rate was determined by referring to the Adler classification standard, wherein, there is insufficient blood flow in Grade 0 and Grade I, but sufficient blood flow in Grade II and Grade III [5].

Magnetic resonance imaging (MRI) equipment used was a Philips Achieva 1.5 T MRI scanner with a dedicated bilateral breast coil. Patients were placed in the prone position, and both breasts were naturally suspended inside the hole in the center of the coil, without compression. The patients were asked to breathe softly and remain motionless. A venous channel was established in advance in the upper arm of each patient, and a long indwelling catheter with a three-way junction was implanted. The two other ends of the three-way junction were connected to 20 ml normal saline and 0.1 mmol/L/Kg dose of contrast agent gadodiamide injection. At the same time, a dynamic scanning sequence was set, and the scanning range covered the entire breast. Six DCE-MRI scans were performed, each lasting for 90 s. First, the masking film was scanned (the first scan). The patient was asked to remain in the original position, and the interval between each scan was 30 s. After the scanning was suspended, a bolus of contrast agent (injected at a speed of 2.5 ml/s, and then 20 ml bolus of normal saline) was injected using a high-pressure syringe. After the suspension was concluded, the scans were performed five times consecutively. The duration of each 3D dynamic sequence scan was 90 s, and the total duration of dynamic enhancement was 9 min. The maximum diameter line and three-dimensional volume of the tumor body were measured using DCE-MRI and magnetic resonance diffusion-weighted imaging (DWI), respectively, and reflected the size and volume of the lesion. On DCE-MRI, the region of the tumor with obvious enhancement (avoiding the necrotic area) was selected as the region of interest, and a time–intensity curve (TIC) was drawn. TIC is an indicator reflecting the microvascular density and vascular permeability of the tissue, and is divided into three types: type I is the inflow type, that is during the dynamic acquisition time, the signal curve shows a continuous and slow increase; type II is the platform type, that is during the dynamic acquisition time, the signal curve begins to rise significantly, and then remains flat; and type III is the outflow type, that is during the dynamic acquisition time, the signal curve begins to rise significantly, and then decreases significantly. DWI was used to analyze the tissue structure and internal characteristics based on the diffusion of water molecules in tissue cells. This method not only provides morphological information of the tumor but also helps evaluate the efficacy by measuring the apparent diffusion coefficient (ADC) of the tumor before and after chemotherapy.

### Triple evaluation method

In this study, we combined physical examination, color Doppler ultrasound, and MRI to evaluate neoadjuvant therapies; the combined method is referred to as triple the evaluation method. Baseline evaluation was conducted before treatment. The efficacy was evaluated by physical examination and color Doppler every cycle, and the efficacy was evaluated by physical examination, color Doppler, and MRI every two cycles. Specific evaluation methods were as follows: (1) complete response (CR): ① all the target lesions disappeared completely under physical examination, color Doppler ultrasound, and MRI; ② the mass was visible under color Doppler ultrasound or MRI; however, the time signal curve of MRI changed from outflow or inflow to the platform, and it repeated continuously twice. (2) Partial response (PR): ① physical examination and imaging examination indicated tumor shrinkage; ② there was no change in tumor size, and one of the following conditions was met: A. the tumor became softer on physical examination than that in the previous cycle; B. the ultrasonic echo changed from a low echo to a leaning low echo and equal echo; C. the blood flow signal of the ultrasonic image changed from rich to medium, small, and then to no blood flow signal; D. MRI enhancement degree decreased; E. MRI time signal curve changed from outflow to inflow to platform. (3) Progressive disease (PD): ① the tumor is enlarged; ② there is no change in the tumor size and one of the following conditions is met: on physical examination, the tumor has become harder, the ultrasonic blood flow is richer than that in the previous cycle, the intensity of MRI has increased, and the time blood flow curve changes from inflow or outflow to the platform. (4) Stable disease (SD): the lesion size showed no change between that of PR and PD.

### Pathological evaluation

According to the Miller–Payne (MP) classification standard for pathological response, the pathological sections obtained after operation were compared with the histological sections obtained after puncture before chemotherapy. Based on the effective rates obtained in previous studies, the major histological response (MHR) groups [6, 7] were classified as Grade 3, 4, and 5, and the non-major histological response (NMHR) groups were classified as Grade 1 and 2. We analyzed the triple evaluation method to determine whether the pathological response was effective/ineffective.

### Statistical analysis

Statistical analysis was performed using SPSS20.0. The measurement data were expressed as $\overline{x}\pm s$, and the counting data were expressed as frequency (%). The age had an approximately normal distribution, which was $\overline{x}\pm s$, and the non-normal distribution was expressed as M (P25, P75). Multiple logistic regression analysis was performed with the classification and grouping of postoperative pathological reactivity of MP grade 3 as the gold standard. The *b* value was used to judge the influence of factors on the final pathology, and the OR value was used to judge whether it was a protective factor or a risk factor. The consistency between the efficacy evaluated using the triple evaluation method and the efficacy evaluated by the gold standard 2 classification of pathology was analyzed using the Kappa test, and the difference was considered statistically significant if *ρ* < 0.05.

## Results

### General clinical data

**Fig. 1:**
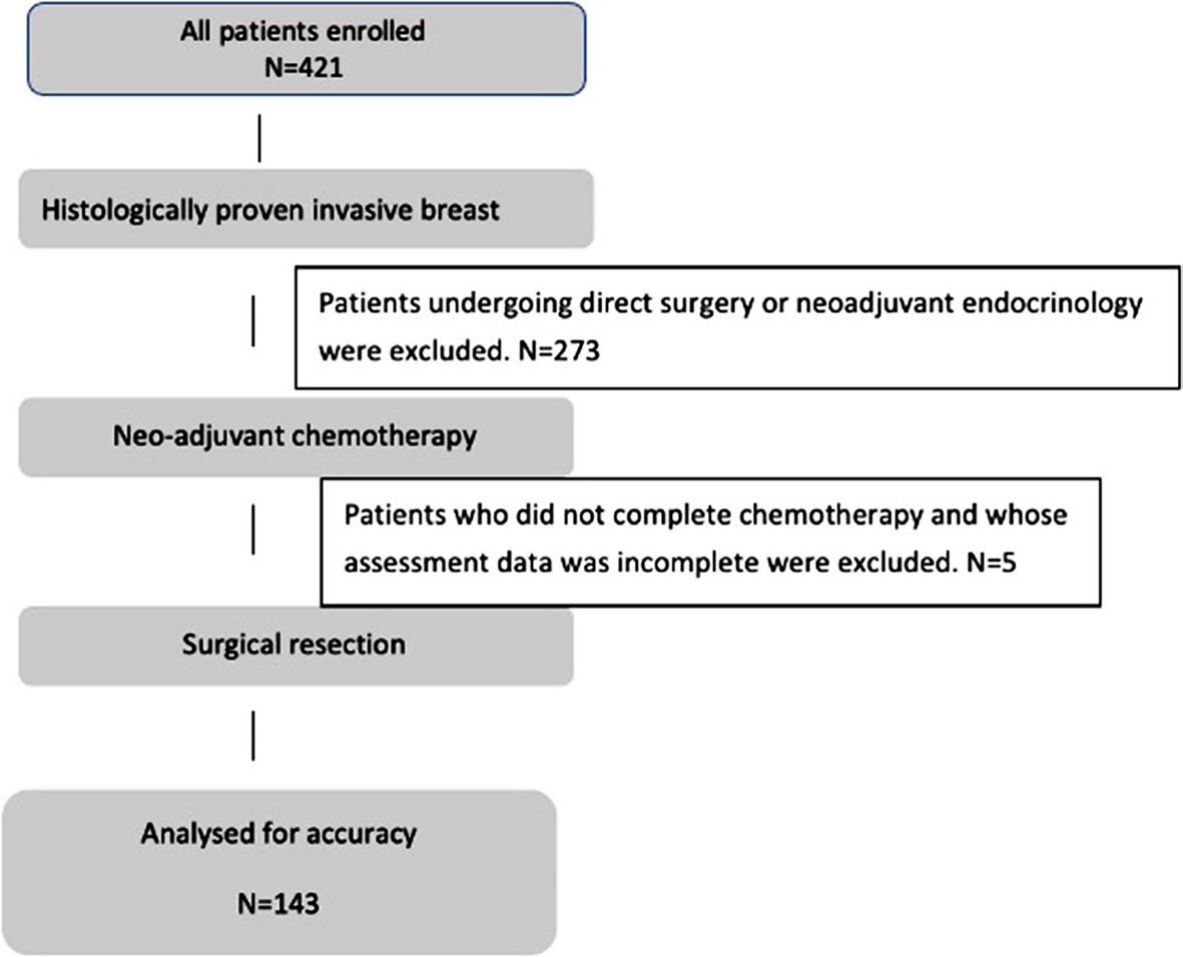
CONSORT diagram

The case files of 143 patients aged 27–73 years with complete clinical data were collected from January 2018 to April 2022. There were 77 premenopausal and 66 postmenopausal women. Molecular typing revealed that 72 cases were hormone receptor-positive, 40 cases were triple-negative breast cancer, and 62 cases were HER2 positive breast cancer. The complete response rate of neoadjuvant therapy was 48.25% (69/143), defined as CR + PR/total = 92.31% (132/143) based on the objective response rate (ORR) previously studied (Table 1). A CONSORT diagram is shown in Fig. 1.

**Table 1:** Patient characteristics

	All patients	Basal-like	HER2+/ER-	Luminal B	Luminal B(HER2+)	Luminal A
*N* (%)	143	40	30	32	32	9
Median age (range)	50 (27,73)	50 (28,70)	54 (36,73)	49 (27,61)	47 (32,68)	47 (35,64)
T1	36	11	8	7	10	0
T2	91	24	20	22	19	6
T3	16	5	2	3	3	3
Stage II	109	36	21	20	25	7
Stage III	34	4	9	12	7	2
Premenopause	77	20	13	17	22	5
Postmenopausal	66	20	17	15	10	4
Chemotherapy successful cases	132	35	30	28	30	9
Chemotherapy failure case	11	5	0	4	2	0

Stage II includes T1N1M0, T2N0M0, T2N1M0, T3N0M0

Stage III includes T1N2M0, T2N2M0, T3N1M0, T3N2M0, T4N0M0, T4N1M0, T4N2M0,T (1-3) N3M0

### Analysis of related factors between clinical and postoperative pathology

We used multiple logistic regression analysis to analyze the correlation between the clinical evaluation parameters and final pathology. The *b* value was used to judge the influence of factors on postoperative pathology and the OR value was used to judge whether the research factor was a protective factor or a risk factor. Based on the analysis, the increase in ultrasonic blood flow after treatment had an impact on pathology; the value of *b* was 2.561 and the value of OR was 12.949, which is a risk factor for failure of chemotherapy. The presence of two preoperative time–signal intensity curves is a therapeutically effective protective factor for inflow. The value of b was −2.622 and the value of OR was 0.073, which is a protective factor for effective chemotherapy (Table 2).

**Table 2:** Physical examination, color Doppler ultrasound, MRI, and pathological effective multivariate logistic regression analysis

	MP grading	B	SE	Wald	*ρ*	OR	95% confidence interval	
							Lower limit	Upper limit
Clinic	The tumor is reduced during palpation.	-3.082	1.805	2.914	.088	.046	.001	1.578
	There is no change in the size of tumor during palpation.	-2.903	2.245	1.672	.196	.055	.001	4.468
	The tumor is soft during palpation.	1.272	.813	2.445	.118	3.567	.725	17.565
	The tumor is hard during palpation.	22.247	2.838	61.438	.000	4,591,041,665.454	17,616,881.500	1,196,446,917,928.918
Ultrasound	The tumor is reduced based on ultrasound.	2.292	1.754	1.708	.191	9.891	.318	307.591
	There is no change in the size of tumor based on ultrasound.	2.111	2.252	.879	.348	8.260	.100	682.047
	The tumor blood flow shows progress based on ultrasound.	2.561	1.285	3.971	.046	12.949	1.043	160.741
	The tumor blood flow does not reduce based on ultrasound.	.582	.619	.884	.347	1.790	.532	6.022
	The preoperative curve is double inflow type.	-2.622	.891	8.658	.003	.073	.013	.417
MRI	The tumor is reduced based on MRI.	.774	.738	1.103	.294	2.169	.511	9.206
	There is no change in the size of tumor based on MRI.	.774	.957	.655	.418	2.169	.333	14.145

*MP* Miller–Payne grading system, pathologic evaluation of neoadjuvant therapy for breast cancer, *B* regression coefficient, *SE* standard error

Further analysis of the correlation between molecular typing analysis and the efficacy of neoadjuvant chemotherapy revealed that HER2-positive tumors are more likely to involve chemotherapy-effective cases and luminal B tumors are more likely to involve chemotherapy-ineffective cases, which deserves clinical attention. Further details are provided in Table 3.

**Table 3:** Multivariate logistic regression analysis to analyze the relationship between molecular typing and pathological outcome

MP classification	*B*	SE	Wald	*df*	Significance level	OR	95% confidence interval	
							Lower limit	Upper limit
Luminal type A	1.825	.980	3.466	1	.063	6.202	.908	42.354
Luminal type B	1.914	.734	6.796	1	.009	6.779	1.608	28.577
Luminal B (HER2-positive type)	-.164	.735	.050	1	.823	.849	.201	3.587
HER2-positive type	-1.954	.986	3.924	1	.048	.142	.021	.980
Triple negative type	0^b^	.	.	0	.	.	.	.

^b^As this parameter is redundant, it is set to zero

### Effectiveness analysis of triple evaluation method

The triple evaluation method can be divided into four categories; however, in this study, we mainly evaluated the relationship between clinical evaluation and pathological effectiveness and ineffectiveness. Therefore, referring to the ORR rate calculation, clinical evaluation of CR and PR was classified as effective, SD was defined as stable, and PD was defined as progress, and the assigned values are 0, 1, and 2, respectively. Valid values for pathology were 0 and non-valid values were 1. The Kappa test was used to analyze the consistency between the clinical evaluation of efficacy and actual effectiveness of the pathological gold standard. The Kappa test result was 0.716 (*p* < 0.05, indicating a statistically significant difference) (Table 4). Thus, the triple evaluation method was effective.

**Table 4:** Consistency analysis of triple evaluation method and pathological gold standard

Clinical efficacy discrimination × two pathological classifications cross table					
Count					
		Two pathological classifications			Total
			0	1	
Clinical efficacy discrimination		69	0	0	69
	2	0	0	5	5
	1	0	24	3	27
	0	0	109	2	111
Total		69	133	10	212
Symmetry measurement					
		Value	Asymptotic standard error^a^	Approximate *T*^b^	Gradual significance
Protocol measurement	Kappa	.716	.039	14.059	.000
Number of valid cases		212			

^a^Not assumed original hypothesis

^b^Asymptotic standard error is used under the assumed original hypothesis

## Discussion

In this paper, we only discuss imaging evaluation methods, wherein, MRI mainly reflects the efficacy of NAT in breast cancer based on the characteristics of lesion morphology and size, dynamic contrast-enhanced semi-quantitative or quantitative parameters, ADC values, and magnetic resonance spectroscopy (MRS) [16–18]. In addition, due to the tumor cell death and reduced cell density, breast cancer patients who are in remission after NAT also present with reduced DWI signals and increased ADC values [19]. At present, multi-parametric MRI (mpMRI) imaging omics lack unified criteria for the selection of different sequence omics, which limits its technical promotion [20, 21]. The traditional mono-exponential model diffusion-weighted imaging (mono-exponential-DWI) is an imaging method for calculating the voluntary movement of water molecules in the tissue gap using a single exponential function. ADC is the most commonly used clinical parameter, reflecting the degree of tissue limitation. The pathological mechanism of applying ADC values to breast tumors is that the proliferation of tumor cells leads to an increase in cell numbers, disorder of tissue structure, and reduction of extracellular space, resulting in the limitation of water molecule movement in the intercellular space of the tissue, increase in DWI signals, and decrease in ADC values. Therefore, there was a significant correlation between ADC values and cell density. At present, however, the correlation between different molecular subtypes and ADC values remains unclear. However, according to several studies, the ADC values of different molecular subtypes are different [22–27], and there is no consensus on the best *b* value of breast DWI. This study further verified the predictive effect of enhanced MRI findings on the outcomes of neoadjuvant therapy. In this study, we found that the therapeutic effect of neoadjuvant therapy can be better evaluated by combining clinical physical examination, color ultrasound, and nuclear magnetic resonance evaluation. The three methods complement each other to avoid the insufficient evaluation of a single method, which is convenient for the majority of prefecty-level hospitals to carry out. In addition, the method is simple and feasible and suitable for promotion.

Morphology is the dominant factor in evaluating the efficacy of breast ultrasound. Thus, it is difficult to distinguish between interstitial fibrosis and residual cancer caused by NAT, and the sensitivity and specificity of pCR prediction for breast cancer are 60.8% and 78%, respectively [28]. Ultrasonic elastography can be used to reflect tissue hardness, and the treatment effect can be reflected by changes in hardness during treatment. Based on the analysis of adipose tissue as a reference, the sensitivity of ultrasonic elastography can reach 80% and the specificity can reach 68% [29], and it can be used to evaluate new auxiliary efficacy. To date, several studies have proved that CEUS can be used to evaluate the efficacy of chemotherapy, and its efficacy in evaluating neoadjuvant chemotherapy is close to that of MRI [30]. However, at present, CEUS has been used only for a short period of time, and its advantages over MRI are not obvious, hence clinical development is limited [31]. Therefore, we selected the ultrasonic blood flow as an evaluation index. This is because after neoadjuvant chemotherapy, the blood vessels in the tumor sensitive to chemotherapy drugs showed atrophy, occlusion, degeneration, and necrosis of tumor cells, which changed the blood flow grading pattern in the tumor. At present, studies have proven that high-frequency color Doppler ultrasound can not only measure the change in breast cancer mass size before and after neoadjuvant chemotherapy but also detect the distribution of blood vessels and hemodynamic changes of microvessels in the lesion, thus providing an objective basis for clinical efficacy evaluation [32], which coincides with the conclusion of the multivariate analysis in this study.

## Conclusions

The triple evaluation method that combines clinical physical examination, color Doppler ultrasound, and MRI proposed in this study can provide a basis for the efficacy evaluation of neoadjuvant chemotherapy for breast cancer and guide prompt treatment scheme continuation or change. This method can ensure that patients undergo the appropriate treatment course and that there is a check on disease progression, and it has a certain clinical application value. In addition, the method is simple and feasible and can be promoted, and more patients can benefit from this relatively economic and pragmatic evaluation method. In the future, with the collection and collation of more clinical data, a scoring system can be established to better guide clinical practice.

## Data Availability

The data that support the findings of this study are available from the corresponding author, upon reasonable request.

## Abbreviations

pCR: Pathologic complete response
EFS: Event-free survival
OS: Overall survival
RECIST: Response evaluation criteria in solid tumors
DCE-MRI: Dynamic contrast-enhanced magnetic resonance imaging
MRI: Magnetic resonance imaging
US: Ultrasound
DWI: Diffusion weighted imaging
TIC: Time–intensity curve
ADC: Apparent diffusion coefficient
CR: Complete response
PR: Partial response
PD: Progressive disease
SD: Stable disease
MP: Miller–Payne
NMHR: Non-major histological response
MHR: Major histological response
pCR: Pathologic complete response
ORR: Objective response rate
NAT: Neoadjuvant therapy
NAC: Neoadjuvant chemotherapy
mpMRI: Multiparametric MRI
CT: Computed tomography
PET: Positron emission tomography
CEUS: Contrast-enhanced ultrasound
US-DOT: Ultrasound diffuse optical tomography
UST: Ultrasound tomography

## References

[1] Cortazar P, Zhang L (2014). Pathological complete response and long-term clinical benefit in breast cancer: the CTNeoBC pooled analysis. [J]. Lancet.

[2] China Breast Cancer neoadjuvant Therapy Expert Group. Expert consensus on neoadjuvant treatment of breast cancer in China (2021 edition). China Oncol. 2022;32(1):80–89. 10.19401/j.cnki.1007-3639.2022.01.011.

[3] Chen M, Zhan WW, Han BS, Fei XC, Jin XL, Chai WM, Wang DB, Shen KW, Wang WP (2012). Accuracy of physical examination, ultrasonography, and magnetic resonance imaging in predicting response to neo-adjuvant chemotherapy for breast cancer. Chin Med J.

[4] Kato E, Mori N, Mugikura S, Sato S, Ishida T, Takase K (2021). Value of ultrafast and standard dynamic contrast-enhanced magnetic resonance imaging in the evaluation of the presence and extension of residual disease after neoadjuvant chemotherapy in breast cancer. Jpn J Radiol.

[5] Matsuda N, Kida K, Ohde S, Suzuki K, Yamauchi H, Nakamura S, Tsunoda H (2018). Change in sonographic brightness can predict pathological response of triple-negative breast cancer to neoadjuvant chemotherapy. Breast Cancer.

[6] Chen M, Wang DB, Fei XC, Tang L, Zhan WW, Wang WP (2013). Predicting response to neoadjuvant chemotherapy in breast cancer by conventional ultrasound and dynamic contrast-enhanced MRI. Chinese J Ultrasound Med.

[7] Martincich L, Montemurro F, De Rosa G, Marra V, Ponzone R, Cirillo S, Gatti M, Biglia N, Sarotto I, Sismondi P, Regge D, Aglietta M (2004). Monitoring response to primary chemotherapy in breast cancer using dynamic contrast-enhanced magnetic resonance imaging. Breast Cancer Res Treat.

[8] Expert Panel on Breast Imaging: Slanetz PJ, Moy L, Baron P, diFlorio RM, Green ED, Heller SL, Holbrook AI, Lee SJ, Lewin AA, Lourenco AP, Niell B, Stuckey AR, Trikha S, Vincoff NS, Weinstein SP, Yepes MM, Newell MS. ACR Appropriateness Criteria® Monitoring Response to Neoadjuvant Systemic Therapy for Breast Cancer. J Am Coll Radiol. 2017;14(11S):S462–S475. 10.1016/j.jacr.2017.08.037. https://pubmed.ncbi.nlm.nih.gov/29101985/. 10.1016/j.jacr.2017.08.037 29101985

[9] Palshof FK, Lanng C, Kroman N, Benian C, Vejborg I, Bak A, Talman ML, Balslev E, Tvedskov TF (2021). Prediction of pathologic complete response in breast cancer patients comparing magnetic resonance imaging with ultrasound in neoadjuvant setting. Ann Surg Oncol.

[10] Samiei S, de Mooij CM, Lobbes MBI, Keymeulen KBMI, van Nijnatten TJA, Smidt ML (2021). Diagnostic performance of noninvasive imaging for assessment of axillary response after neoadjuvant systemic therapy in clinically node-positive breast cancer: a systematic review and meta-analysis. Ann Surg.

[11] Morgan C, Stringfellow TD, Rolph R, Kovacs T, Kothari A, Pinder SE, Hamed H, Sever AR (2020). Neoadjuvant chemotherapy in patients with breast cancer: does response in the breast predict axillary node response?. Eur J Surg Oncol.

[12] Hamy AS, Bonsang-Kitzis H, De Croze D, Laas E, Darrigues L, Topciu L, Menet E, Vincent-Salomon A, Lerebours F, Pierga JY, Brain E, Feron JG, Benchimol G, Lam GT, Laé M, Reyal F (2019). Interaction between molecular subtypes and stromal immune infiltration before and after treatment in breast cancer patients treated with neoadjuvant chemotherapy. Clin Cancer Res.

[13] Cullinane C, Creavin B, O'Leary DP, O'Sullivan MJ, Kelly L, Redmond HP, Corrigan MA (2020). Can the neutrophil to lymphocyte ratio predict complete pathologic response to neoadjuvant breast cancer treatment? A systematic review and meta-analysis. Clin Breast Cancer.

[14] Di Cosimo S, Triulzi T, Pizzamiglio S, De Cecco L, de Azambuja E, Fumagalli D, Putzai L, Harbeck N, Izquierdo M, Peña L, Daidone MG, Huober J, Gori S, Cinieri S, Torri V, Baselga J, Piccart M, de Braud FG, Apolone G, Verderio P, Tagliabue E (2019). The 41-gene classifier TRAR predicts response of HER2 positive breast cancer patients in the NeoALTTO study. Eur J Cancer.

[15] Fu C, Liu Y, Han X, Pan Y, Wang HQ, Wang H, Dai H, Yang W (2021). An immune-associated genomic signature effectively predicts pathologic complete response to neoadjuvant paclitaxel and anthracycline-based chemotherapy in breast cancer. Front Immunol.

[16] Romeo V, Accardo G, Perillo T, Basso L, Garbino N, Nicolai E, Maurea S, Salvatore M (2021). Assessment and prediction of response to neoadjuvant chemotherapy in breast cancer: a comparison of imaging modalities and future perspectives. Cancers (Basel).

[17] Rauch GM, Adrada BE, Kuerer HM, van la Parra RF, Leung JW, Yang WT. (2017). Multimodality imaging for evaluating response to neoadjuvant chemotherapy in breast cancer. AJR Am J Roentgenol.

[18] Ji Y, Zhao R, Lu H, Liu PF (2021). MRI evaluation of neoadjuvant therapy in breast cancer. Chinese J Radiol.

[19] Partridge SC, Zhang Z, Newitt DC, Gibbs JE, Chenevert TL, Rosen MA, Bolan PJ, Marques HS, Romanoff J, Cimino L, Joe BN, Umphrey HR, Ojeda-Fournier H, Dogan B, Oh K, Abe H, Drukteinis JS, Esserman LJ, Hylton NM, ACRIN 6698 Trial Team and I-SPY 2 Trial Investigators (2018). Diffusion-weighted MRI findings predict pathologic response in neoadjuvant treatment of breast cancer: the ACRIN 6698 Multicenter Trial. Radiology.

[20] Zhu XL, Wu JL (2022). Application progress of MRI radiomics in the efficacy and prognosis of neoadjuvant chemotherapy for breast cancer. Magn Reson Imaging.

[21] Lu DM, Wang JM, Liu YL, Yang XP (2018). Progress in the application of different DWI exponential models for breast lesions. Magn Reson Imaging.

[22] Martincich L, Deantoni V, Bertotto I, Redana S, Kubatzki F, Sarotto I, Rossi V, Liotti M, Ponzone R, Aglietta M, Regge D, Montemurro F (2012). Correlations between diffusion-weighted imaging and breast cancer biomarkers. Eur Radiol.

[23] Kim EJ, Kim SH, Park GE, Kang BJ, Song BJ, Kim YJ, Lee D, Ahn H, Kim I, Son YH, Grimm R (2015). Histogram analysis of apparent diffusion coefficient at 3.0t: Correlation with prognostic factors and subtypes of invasive ductal carcinoma. J Magn Reson Imaging.

[24] Kawashima H, Miyati T, Ohno N, Ohno M, Inokuchi M, Ikeda H, Gabata T (2017). Differentiation between luminal-A and luminal-B breast cancer using intravoxel incoherent motion and dynamic contrast-enhanced magnetic resonance imaging. Acad Radiol.

[25] Chen W, Zhang J, Long D, Wang Z, Zhu JM (2017). Optimization of intra-voxel incoherent motion measurement in diffusion-weighted imaging of breast cancer. J Appl Clin Med Phys.

[26] Li W, Newitt DC, Gibbs J, Wilmes LJ, Jones EF, Arasu VA (2020). Predicting breast cancer response to neoadjuvant treatment using multi-feature MRI: results from the I-SPY 2 TRIAL. NPJ Breast Cancer.

[27] Montemezzi S, Benetti G, Bisighin MV, Camera L, Zerbato C, Caumo F, Fiorio E, Zanelli S, Zuffante M, Cavedon C (2021). 3T DCE-MRI radiomics improves predictive models of complete response to neoadjuvant chemotherapy in breast cancer. Front Oncol.

[28] Baumgartner A, Tausch C, Hosch S, Papassotiropoulos B, Varga Z, Rageth C, Baege A (2018). Ultrasound-based prediction of pathologic response to neoadjuvant chemotherapy in breast cancer patients. Breast..

[29] Wang Y, Wang CF, Liu XS, Wang L, Du J, Li FH, Li HL (2017). Value of ultrasonic elasticity score and strain ratio in evaluating breast cancer neoadjuvant chemotherapy. J Clin Ultrasound Med.

[30] Zhang L, Hao J, Wang LP, Shen WZ, Wang D, Yang YL (2014). Contrast-enhanced ultrasound, color doppler ultrasound and MRI-PWI for evaluating the response of breast cancer to neoadjuvant chemotherapy. J Huazhong Univ Sci Technol (Medical Science).

[31] Sedura U, Baiktiar M, Lv XY (2017). Ultrasound study on the effect of chemotherapy in women with breast cancer. Cancer Progression.

[32] Hu ZM, Sun DS, Zhong JY (2013). Role of color Doppler ultrasound in evaluation of breast cancer during neoadjuvant chemotherapy. Chinese J Clin Med Imaging.

